# Supplementary material for: SlicerMorph photogrammetry: an open-source photogrammetry workflow for reconstructing 3D models
Source: Biol Open. 2025 Aug 29;14(8):bio062126. doi: 10.1242/bio.062126 (PMC12421799; doi:10.1242/bio.062126)
Supplement: Supplementary information [file biolopen-14-062126-s1.pdf]

Biology Open (2025): doi:10.1242/bio.062126: Supplementary information

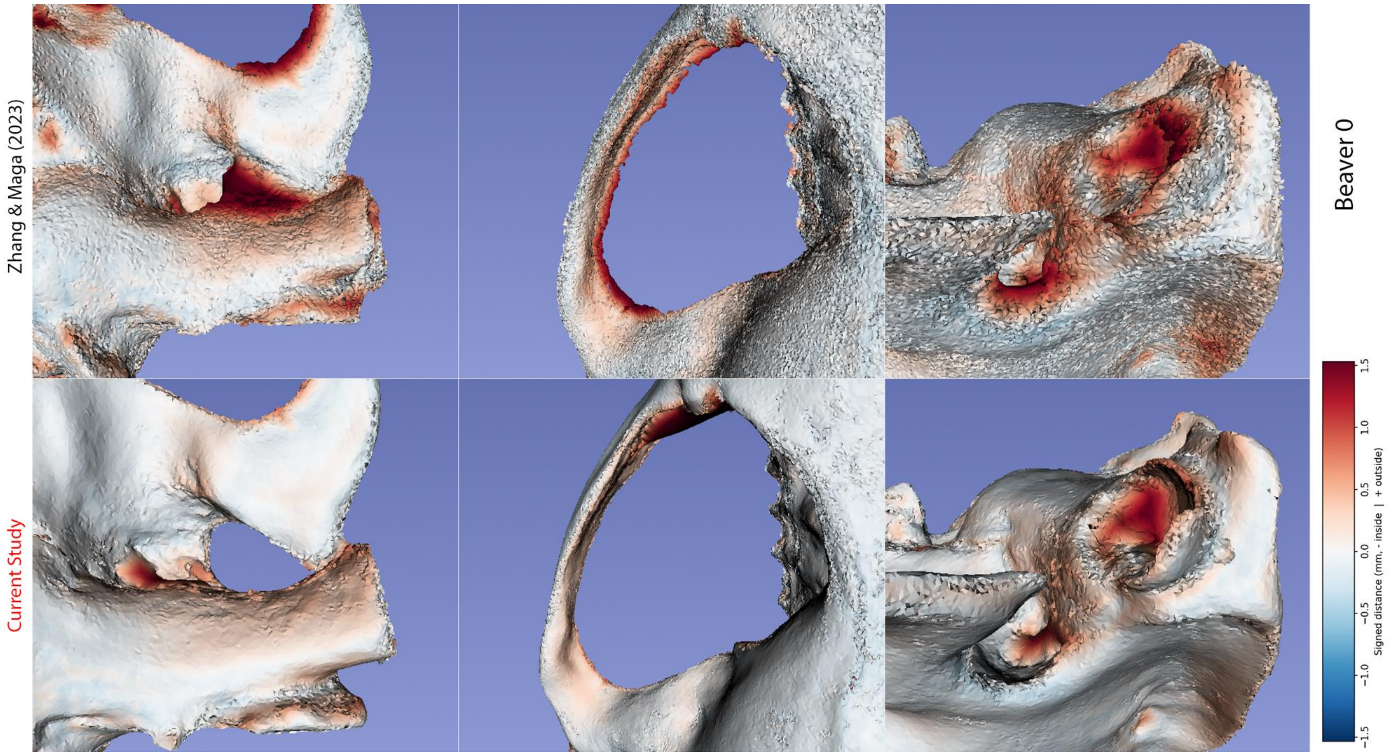

Fig. S1. Higher-fidelity recovery of three fragile cranial regions in an adult mountain beaver (Aplodontia rufa, “Beaver 0”). Surfaces are color-mapped by signed distance to the micro-CT reference (scale at right; warm = outside the true surface, cool = inside). Upper row, original photogrammetry workflow of Zhang & Maga (2023); lower row, refined workflow developed here.

First Column: ventrolateral view of the squamosal complex. The underside of the posterior root of the zygomatic arch is uppermost; the external acoustic (auditory) meatus and adjoining postglenoid process occupy the lower field. The upper model shows pitting, ragged sutures, and large positive deviations (red). The new pipeline yields a smooth, continuous zygomatic root and a sharply bounded meatal rim, with deviations confined to < 0.5 mm.

Second Column: dorsolateral oblique view across the orbit. The camera looks down the long axis of the zygomatic arch, capturing the thin orbital rim and the maxillafrontal suture. The upper legacy model exhibits a thinned arch, with extruded extra polygons along the superior ridge of the zygomatic arch. The revised workflow reconstructs the arch as a full-thickness hoop and removes spurious polygons.

Third Column: ventrolateral view of the otic & basicranial region. The external acoustic meatus is seen in a strict profile; the lateral edge of the pterygoid process is just visible at the far left margin. The 2023 polygon model is riddled with holes and exaggerated bosses; the current workflow restores a coherent tympanic recess and contiguous pterygoid flange, reducing both over- and undershoot artefacts.

Each figure (**Figs S2–S15**) below illustrates the geometric accuracy of the 3D models created using the workflow presented in Zhang and Maga 2023 and from the improved workflow presenting in this study, for every mountain beaver (*Aplodontia rufa*) skull. Each figure depicts dorsal, ventral, and oblique posterior-lateral views of the specimen reconstructed using their respective workflows (Zhang and Maga 2023 on left, current study on right). The heatmaps reflect the magnitude of deviation (in mm) from the corresponding 3D model derived from micro-CT scanning that serves as the ground-truth reference. Warmer colors (red shades) represent areas with higher deviation, whereas lighter colors (yellow shades) indicate regions of low deviation. Across all specimens, results from the current study with the improved workflow consistently show reduced artifacts and improved reconstruction quality, particularly around challenging structures such as orbital margins, zygomatic arches, and foramina. Regardless of the workflow, the highest deviations are always in the openings of nasal cavity, choana and foramen magnum. That’s because in microCT derived ground truth models, these are actual openings into the cranial cavity or nasopharyngeal passages. On the other hand, in photogrammetry models are reconstructed as solid, water-tight structures, hence these openings are closed (stitched over).

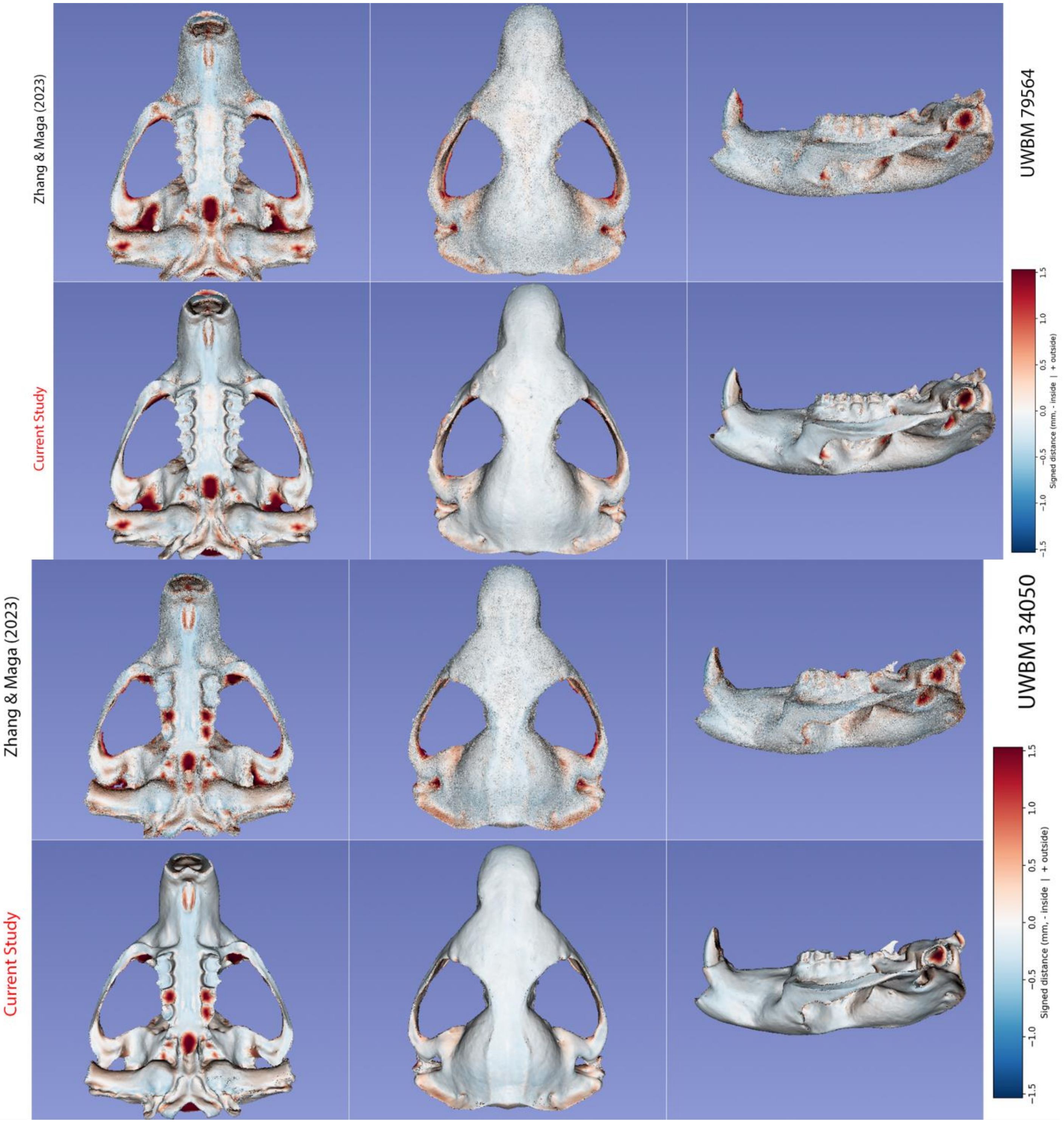
Zhang & Maga (2023)
Current Study
UWBM 79564
Zhang & Maga (2023)
Current Study
UWBM 34050
Signed distance (mm, - inside | + outside)
−1.5 −1.0 −0.5 0.0 0.5 1.0 1.5

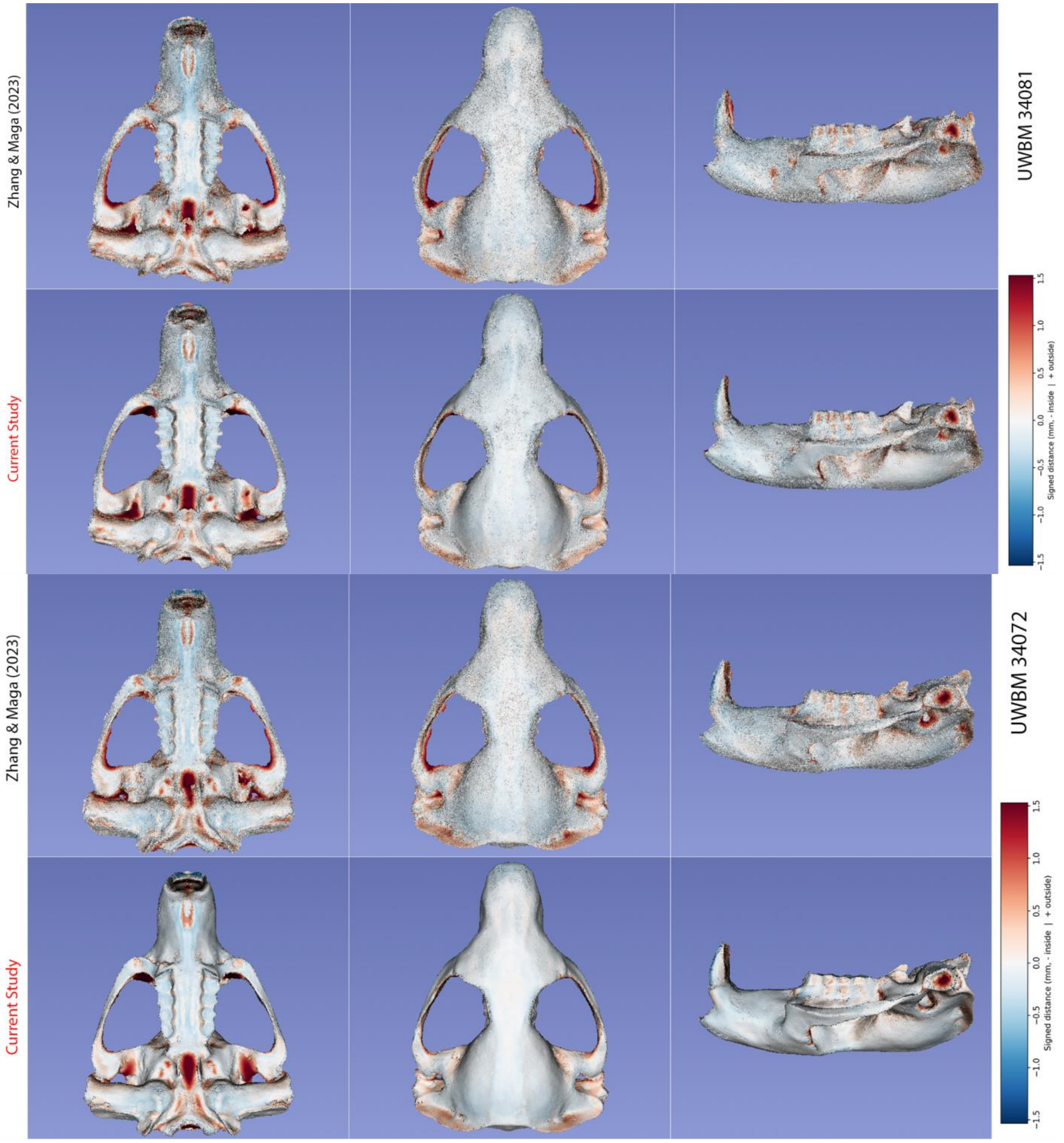

Biology Open • Supplementary information

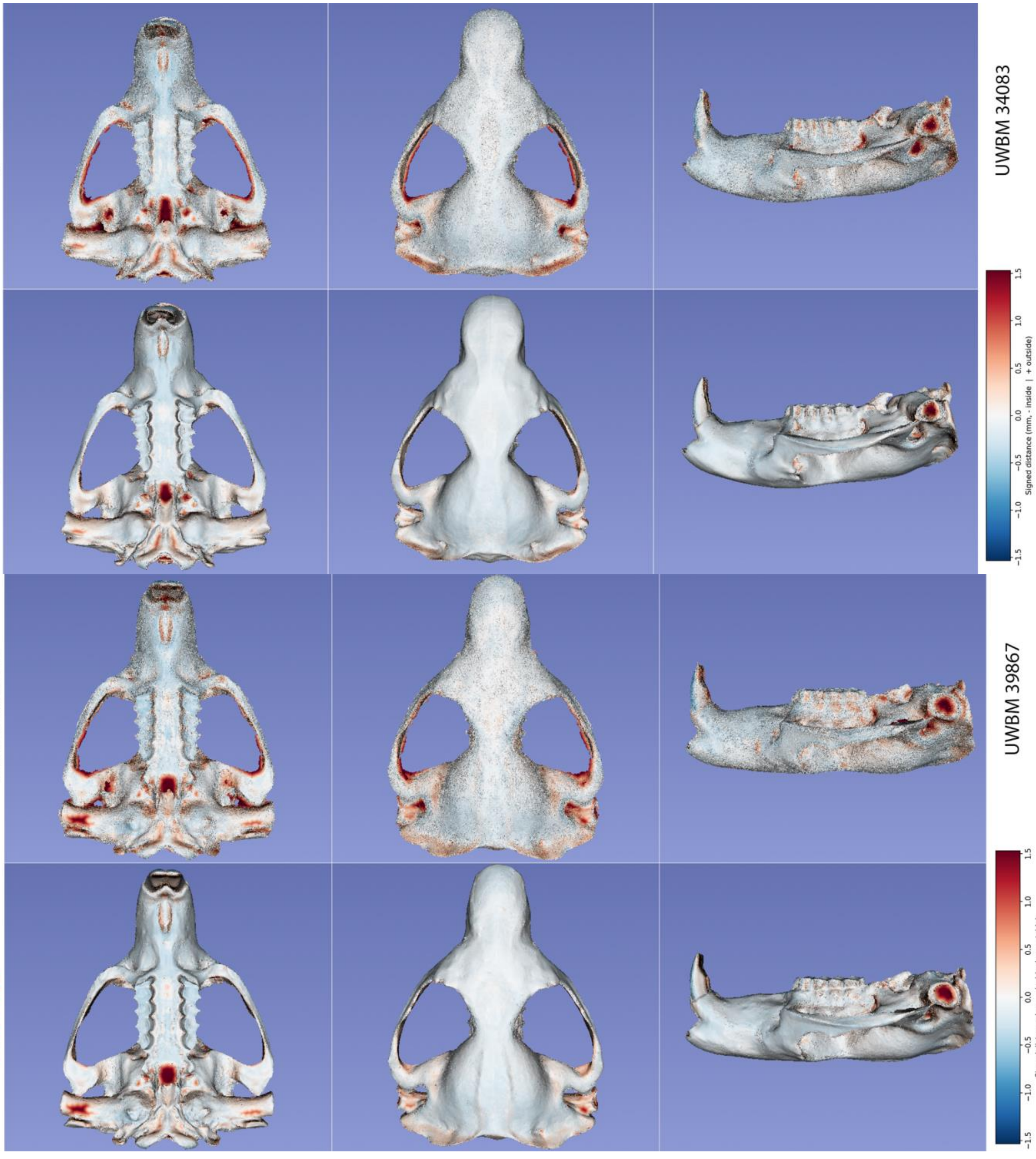

Biology Open • Supplementary information

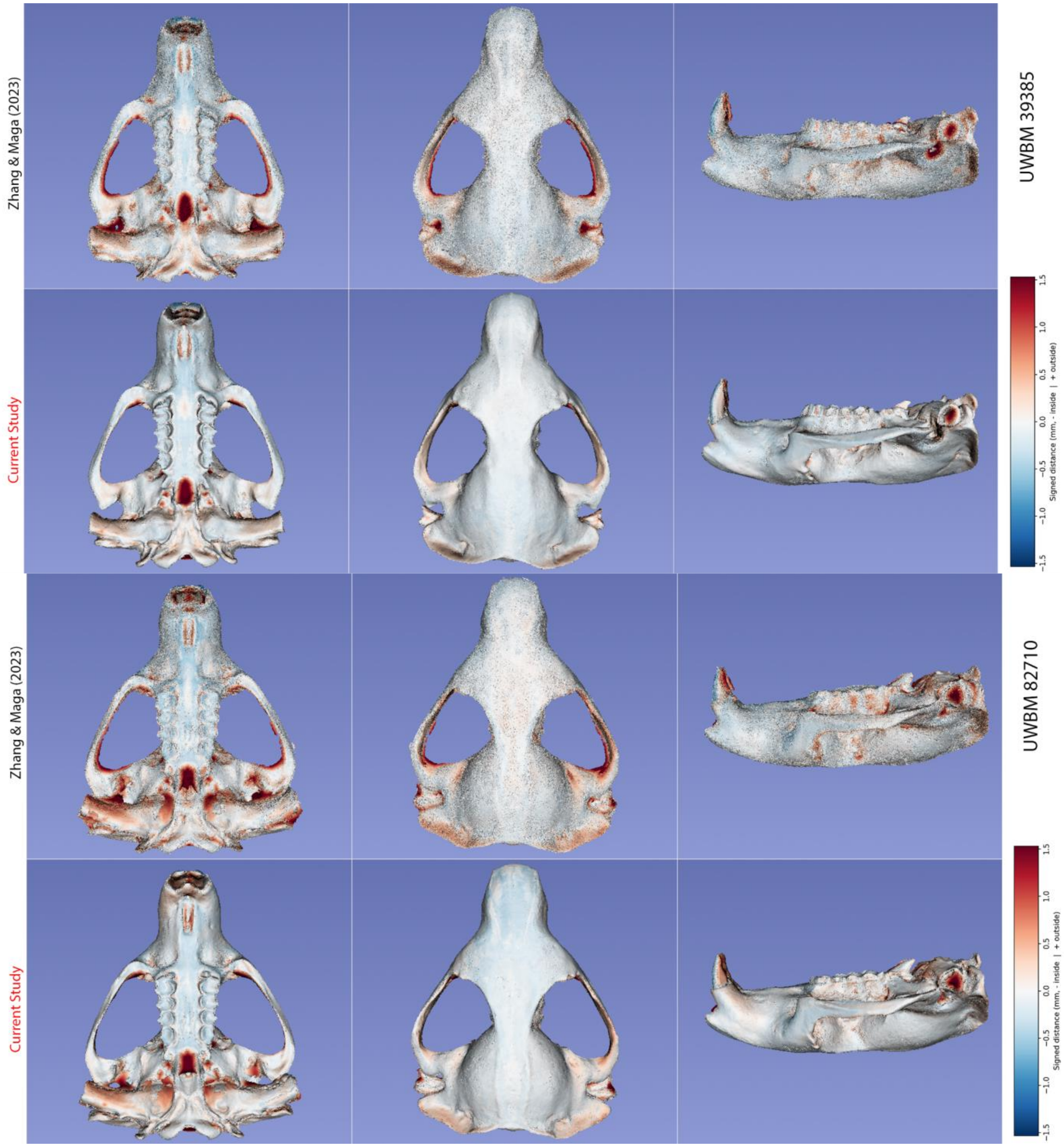
Zhang & Maga (2023)
Current Study
UWBM 39385
Zhang & Maga (2023)
Current Study
UWBM 82710
Signed distance (mm, - inside | + outside)
−1.5
−1.0
−0.5
0.0
0.5
1.0
1.5

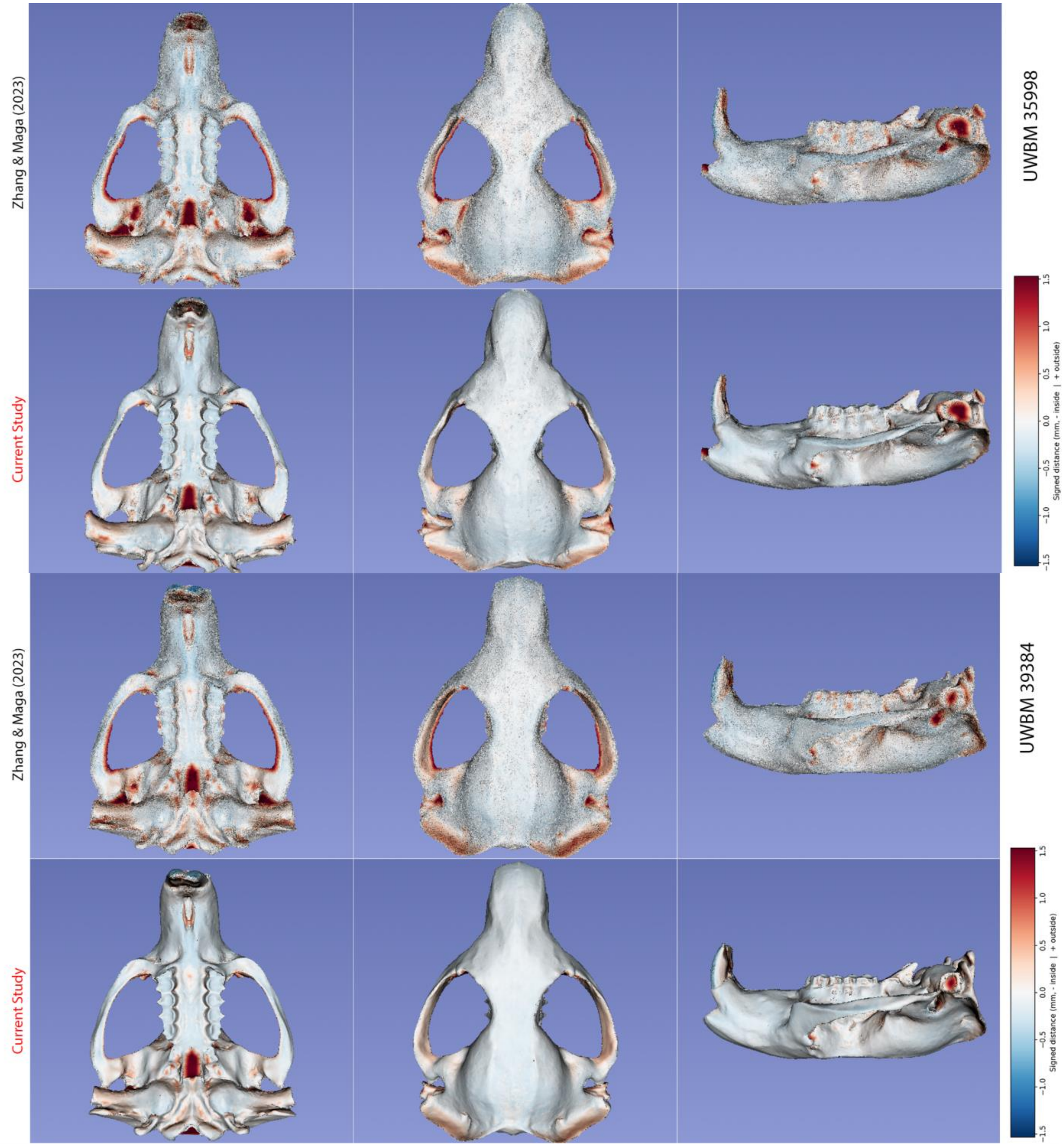

Biology Open • Supplementary information

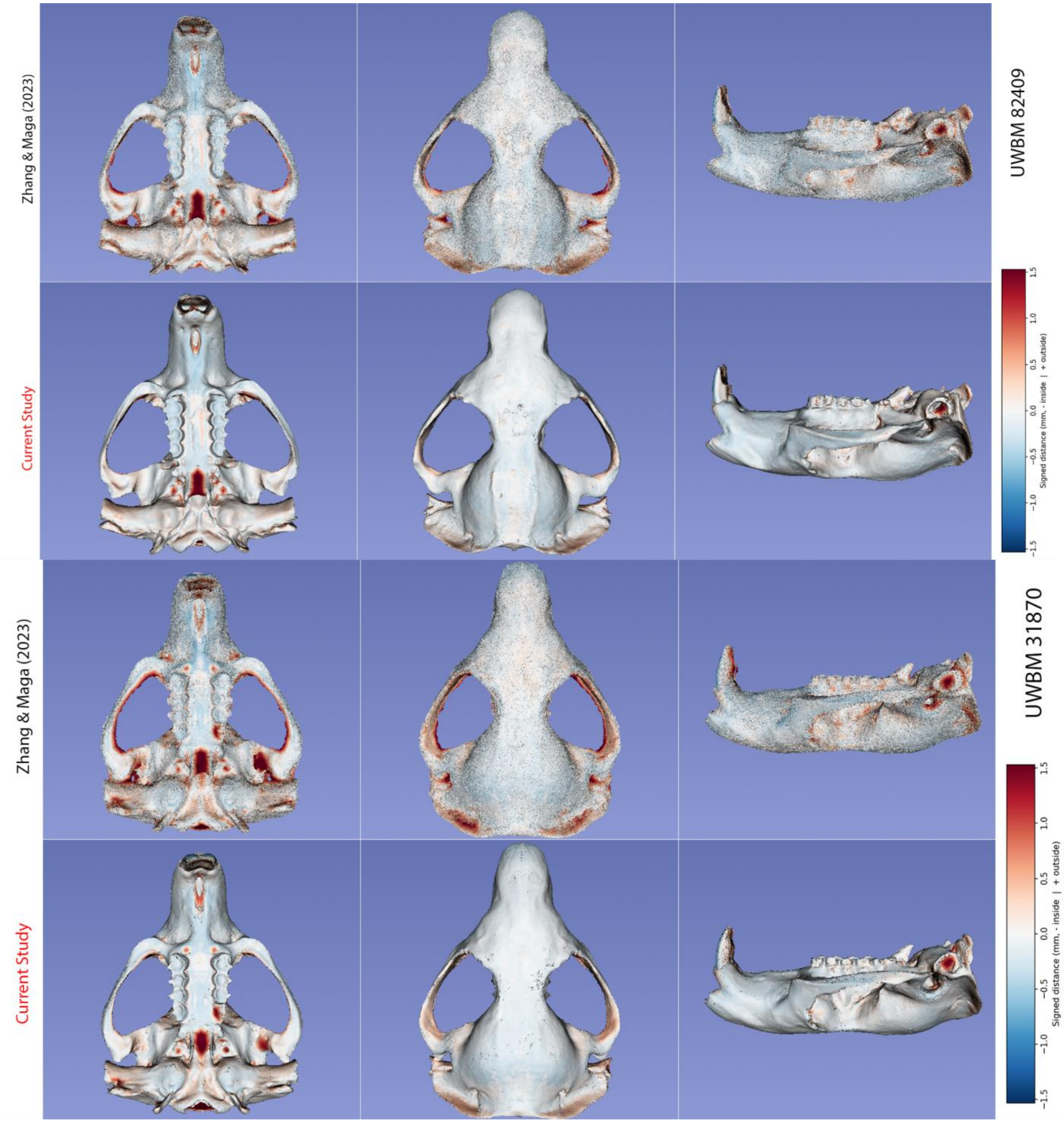
Zhang & Maga (2023)
Current Study
UWBM 82409
Signed distance (mm, - inside | + outside)
−1.5
−1.0
−0.5
0.0
0.5
1.0
1.5
Zhang & Maga (2023)
Current Study
UWBM 31870
Signed distance (mm, - inside | + outside)
−1.5
−1.0
−0.5
0.0
0.5
1.0
1.5

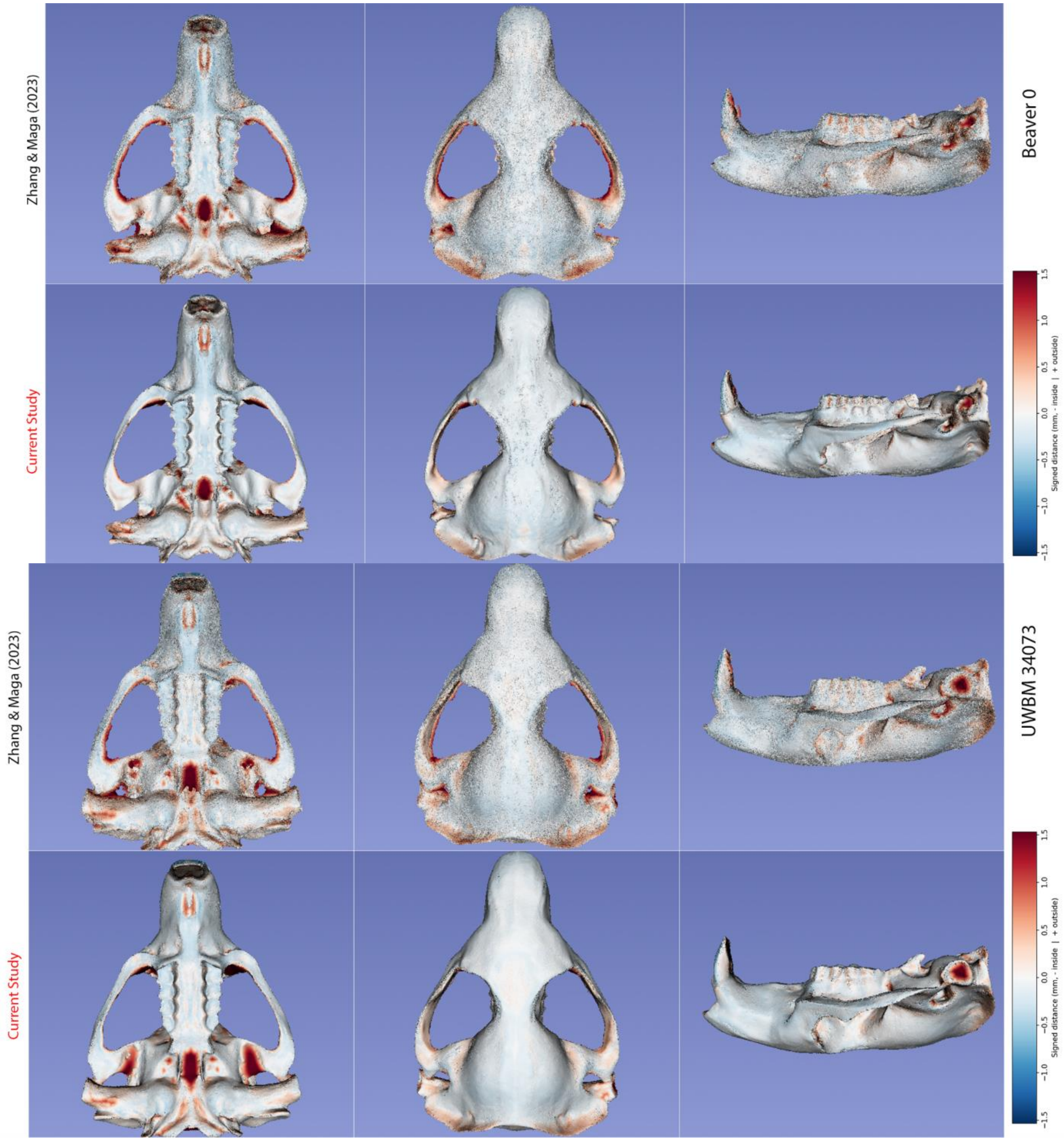

Biology Open • Supplementary information

Table S1. Polygon Model-level statistics for each skull reconstructed with the improved SlicerMorph Photogrammetry workflow (“Current study”) versus the original Zhang & Maga (2023) workflow. Vertex and triangle counts are reported for both pipelines; texture-map dimensions and total reconstruction time are available only for the new workflow. Across the 14 specimens processed with the new pipeline, mean values were 5.73 × 10^5 vertices, 5.40 × 10^5 triangles, and 3,736 s (≈ 62 min) reconstruction time. Note: specimens UWBM 39384 and UWBM 82409 were rerun with higher point density to rescue failed polygon models, resulting in unusually high triangle counts.

Specimen ID	Workflow	Vertices	Triangles	Texture resolution (px)	Reconstruction time (s)
UWBM 31870	Current study	372796	506358	5027 x 5034	6286.23
UWBM 31870	Zhang & Maga 2023	418157	807373		
UWBM 34050	Current study	368804	544772	4776 x 5036	5490.98
UWBM 34050	Zhang & Maga 2023	452206	889131		
UWBM 34072	Current study	427299	550930	4270 x 4275	2971.25
UWBM 34072	Zhang & Maga 2023	431399	840170		
UWBM 34073	Current study	415603	567781	4274 x 4275	4400.94
UWBM 34073	Zhang & Maga 2023	445544	871946		
UWBM 34081	Current study	423131	537838	4273 x 4524	1852.91
UWBM 34081	Zhang & Maga 2023	426422	828587		
UWBM 34083	Current study	384178	524215	5028 x 5034	4731.05
UWBM 34083	Zhang & Maga 2023	453013	885955		
UWBM 35998	Current study	374135	544175	5028 x 5034	2958.28
UWBM 35998	Zhang & Maga 2023	437828	853641		
UWBM	Current study	170158	567195	5027 x 5034	3399.67

39384†		5			
UWBM 39384	Zhang & Maga 2023	450552	885019		
UWBM 39385	Current study	368477	537926	5028 x 5283	3011.61
UWBM 39385	Zhang & Maga 2023	437180	851499		
UWBM 39867	Current study	372623	548629	5029 x 5034	4336.28
UWBM 39867	Zhang & Maga 2023	446287	874994		
UWBM 79564	Current study	368401	538116	4778 x 5034	3681.39
UWBM 79564	Zhang & Maga 2023	452332	888310		
UWBM 82409†	Current study	1689642	563214	5028 x 5283	2092.19
UWBM 82409	Zhang & Maga 2023	456739	897502		
UWBM 82710	Current study	367883	512465	5026 x 5285	3565.23
UWBM 82710	Zhang & Maga 2023	409150	790752		
Beaver 0	Current study	381228	515114	5027 x 5034	3530.29
Beaver 0	Zhang & Maga 2023	436239	849855		

Biology Open • Supplementary information
